# Supplementary figures and images for: Unraveling the Microbiota of Natural Black cv. Kalamata Fermented Olives through 16S and ITS Metataxonomic Analysis
Source: Microorganisms. 2020 May 6;8(5):672. doi: 10.3390/microorganisms8050672 (PMC7284738; doi:10.3390/microorganisms8050672)

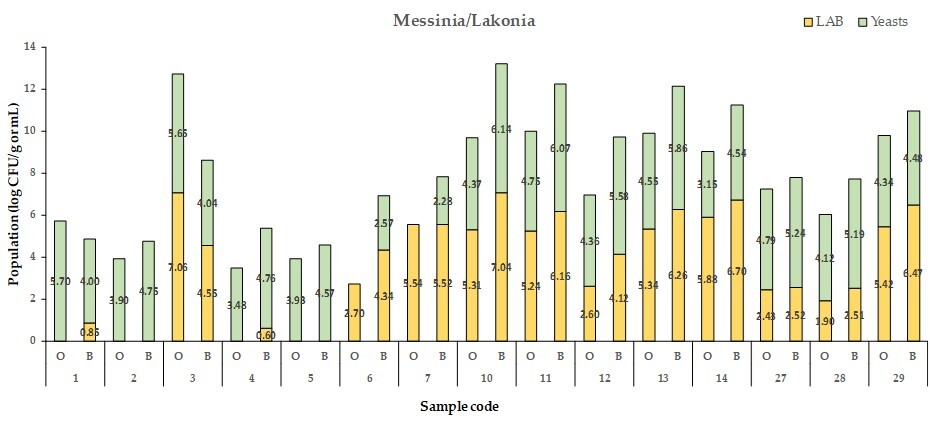

Supplement: Supplementary file 1 [file microorganisms-08-00672-s001.zip › Figure S1.jpg]

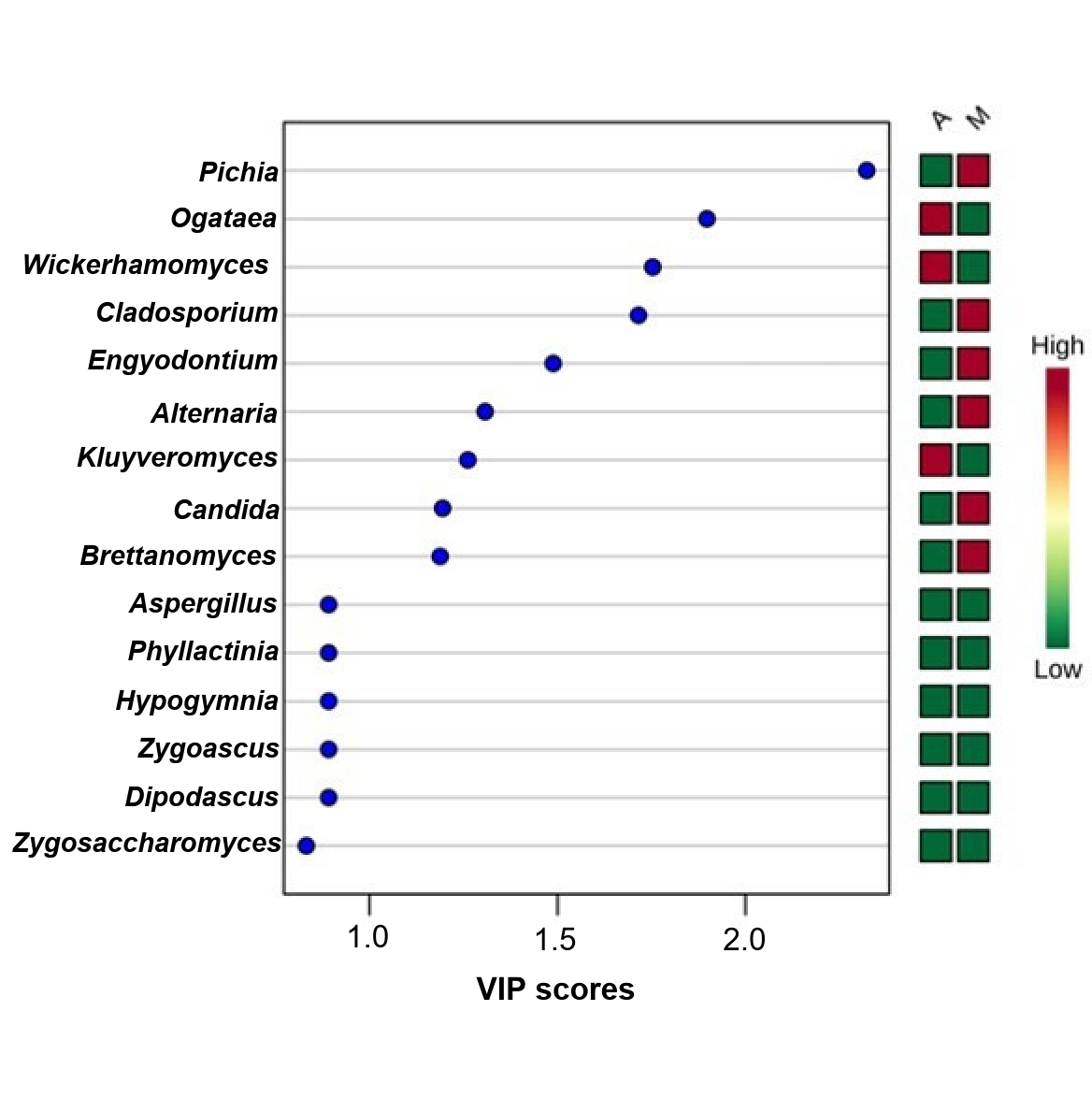

Supplement: Supplementary file 1 [file microorganisms-08-00672-s001.zip › Figure S10.png]

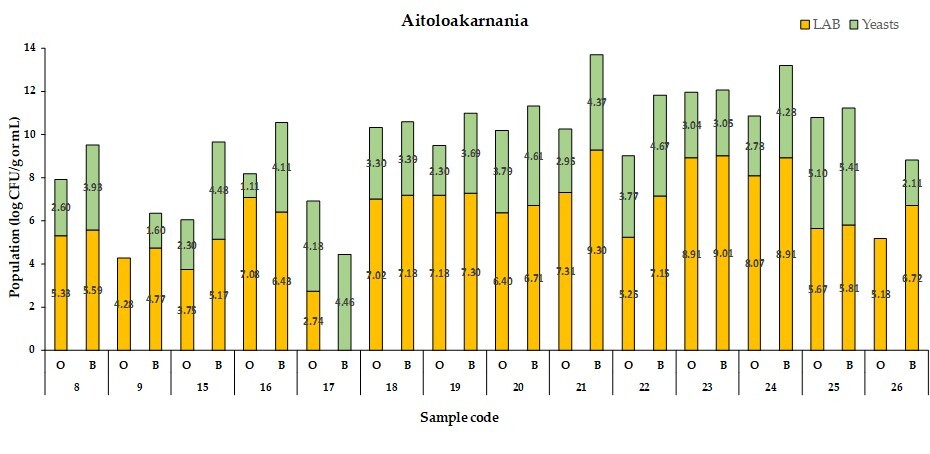

Supplement: Supplementary file 1 [file microorganisms-08-00672-s001.zip › Figure S2.jpg]

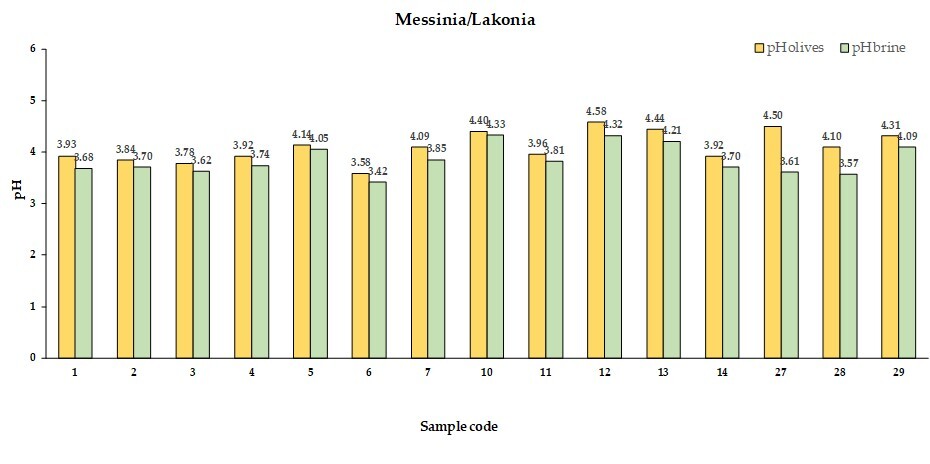

Supplement: Supplementary file 1 [file microorganisms-08-00672-s001.zip › Figure S3.jpg]

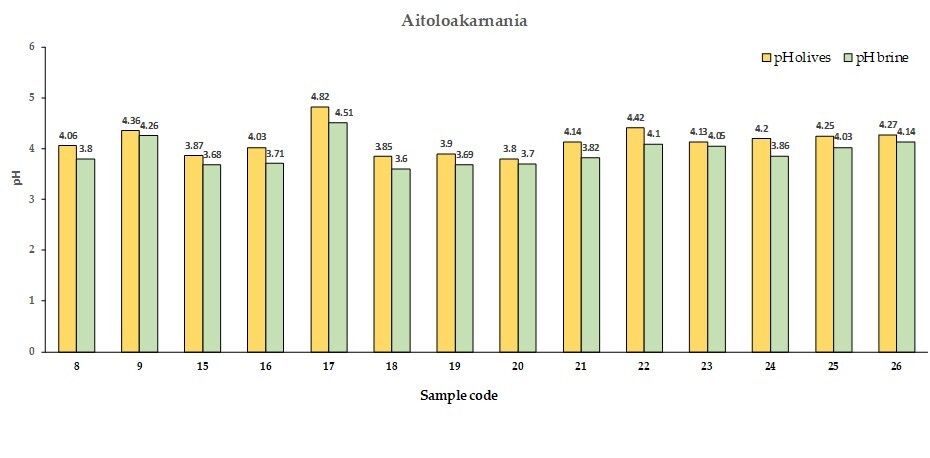

Supplement: Supplementary file 1 [file microorganisms-08-00672-s001.zip › Figure S4.jpg]

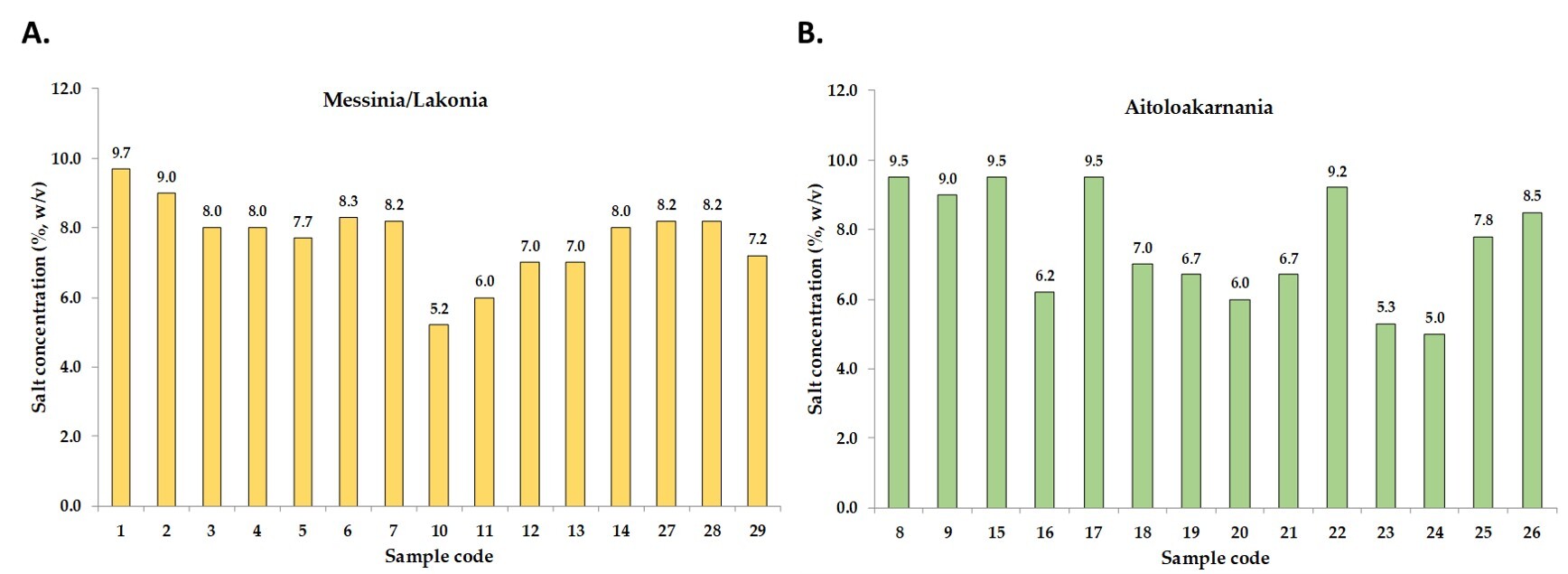

Supplement: Supplementary file 1 [file microorganisms-08-00672-s001.zip › Figure S5.jpg]

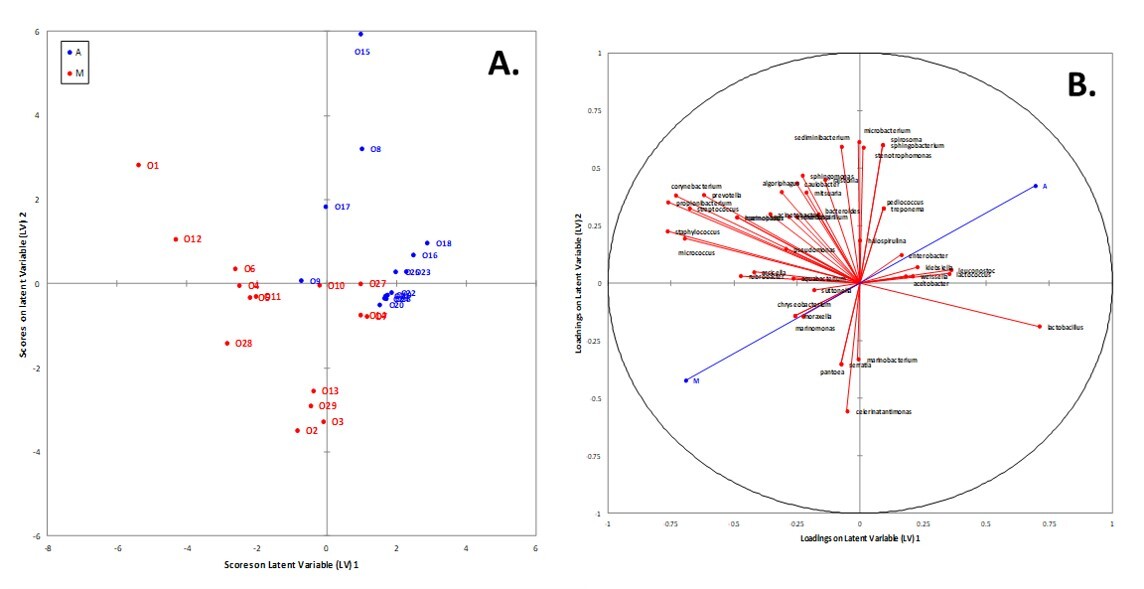

Supplement: Supplementary file 1 [file microorganisms-08-00672-s001.zip › Figure S7.jpg]

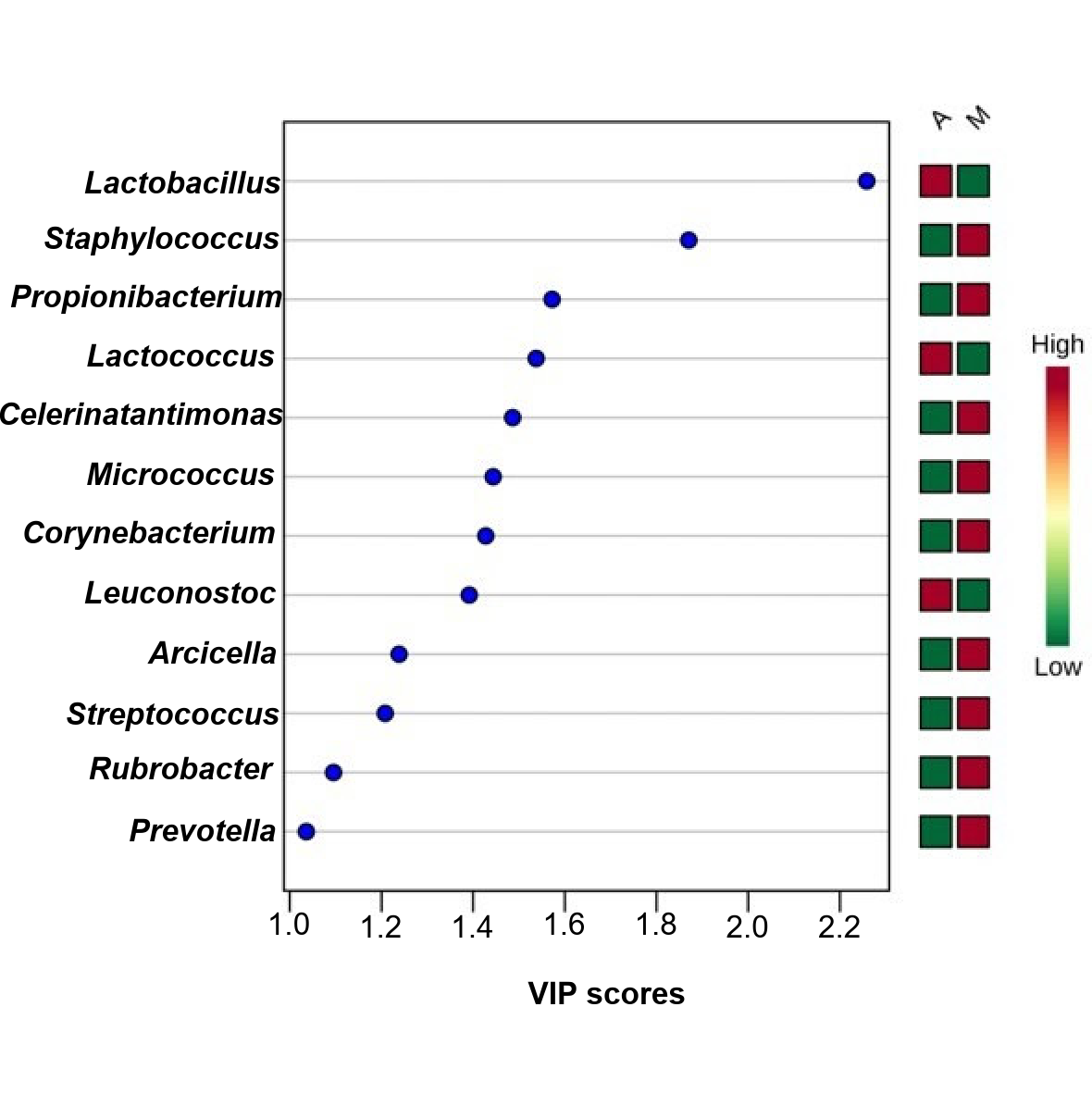

Supplement: Supplementary file 1 [file microorganisms-08-00672-s001.zip › Figure S8.png]

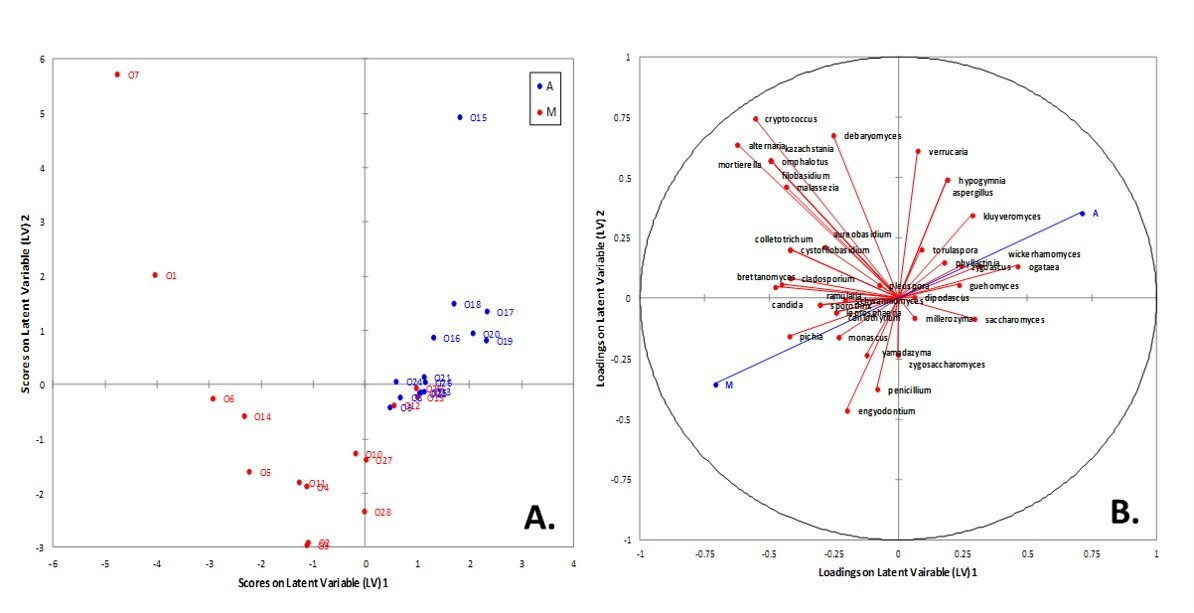

Supplement: Supplementary file 1 [file microorganisms-08-00672-s001.zip › Figure S9.jpg]
